# 4-Hydroxybenzaldehyde accelerates acute wound healing through activation of focal adhesion signalling in keratinocytes

**DOI:** 10.1038/s41598-017-14368-y

**Published:** 2017-10-27

**Authors:** Chan Woo Kang, Ye Eon Han, Jean Kim, Joo Heon Oh, Yoon Hee Cho, Eun Jig Lee

**Affiliations:** 1 Brain Korea 21 PLUS Project for Medical Science, Yonsei University, Seoul, Korea; 2 Endocrinology, Institute of Endocrine Research, College of Medicine, Yonsei University, Seoul, Korea; 3 Department of Biochemistry, Yonsei University, Seoul, Korea

## Abstract

4-Hydroxybenzaldehyde (4-HBA) is a naturally occurring benzaldehyde and the major active constituent of *Gastrodia elata*. While recent studies have demonstrated metabolic effects of 4-HBA, little is known about the physiological role of 4-HBA in acute wound healing. Here, we investigated the effects and mechanisms of 4-HBA on acute wound healing. Using an *in vitro* approach, we found that 4-HBA significantly promoted keratinocyte cell migration and invasion by increasing focal adhesion kinase and Src activity. In addition, 4-HBA treatment also promoted wound healing and re-epithelialization in an *in vivo* excision wound animal model. Combination treatment with 4-HBA and platelet-derived growth factor subunit B homodimer showed synergistic effects in promoting wound healing. Taken together, our results demonstrated that treatment with 4-HBA promoted keratinocyte migration and wound healing in mouse skin through the Src/mitogen-activated protein kinase pathway. Therefore, 4-HBA could be a candidate therapeutic agent with the potential to promote acute wound healing.

## Introduction

Acute wound healing is a regenerative process that maintains the barrier function of the skin after injury, and requires dynamic interaction of various cell types^1^. This process consists of three partially overlapping phases: inflammation, re-epithelialization, and tissue remodelling. During the re-epithelialization step, epidermal cells migrate to the wound site through proliferation and differentiation, and the epidermal barrier is reconstituted. Next, angiogenesis and vasculogenesis promote neovascularization at the wound area. During angiogenesis, major angiogenic factors, such as vascular endothelial growth factor (VEGF) and transforming growth factor (TGF)-β1, are critical for new blood vessel formation^2,3^. Defects in cell migration or angiogenesis often lead to incomplete wound healing^4–7^. Re-epithelialization and neovascularization are important for wound repair; however, few studies have investigated the detailed molecular mechanisms involved in this process.

Medicinal plants, particularly their active compounds, have long been used to promote wound repair^8^. Furthermore, the use of alternative therapies and natural remedies for rapid wound healing is on the rise^9^. Benzaldehyde is an organic compound designated as “generally recognized as safe (GRAS)” by the United States Food and Drug Administration (FDA) and is used industrially as a flavouring agent and fragrance^10^. Recent studies have reported the therapeutic effects of benzaldehydes in diseases such as cancer, vascular disease, and renal disease^11–14^. 4-Hydroxybenzaldehyde (4-HBA) is an active compound isolated from *Gastrodia elata* (Tianma), which has long been used as a Chinese herbal medicine to treat headaches, migraines, as well as some neuralgias and nervous disorders^9,15^. 4-HBA is a structural isomer of salicylaldehyde with one hydroxyl (-OH) group at the *para* position of the phenolic ring. Several studies have suggested that 4-HBA is an active candidate for improving insulin resistance and inhibiting cholinesterase^16,17^. However, the therapeutic effects of 4-HBA on acute wound healing have not been determined.

We previously reported the effects of 3-HBA and 4-HBA on atherosclerosis^18^. *In vivo* Matrigel plug assays showed that 3-HBA had vasoprotective effects, whereas 4-HBA had angiogenesis-promoting effects. Accordingly, in this study, we investigated the effects of 4-HBA on acute wound repair processes both *in vitro* and *in vivo*. Additionally, we tested a combination treatment of 4-HBA with platelet-derived growth factor subunit B homodimer (PDGF-BB), which is approved by the US FDA for the treatment of wound closure and diabetic limb ulcers^19^. Finally, we examined the wound healing effects of 4-HBA on the dorsal skin of C57BL6 mice. Overall, our findings provided important insights into the potential clinical applications of 4-HBA for the acceleration of wound healing.

## Results

### 4-HBA induced the migration of HaCaT cells

**Figure 1:**
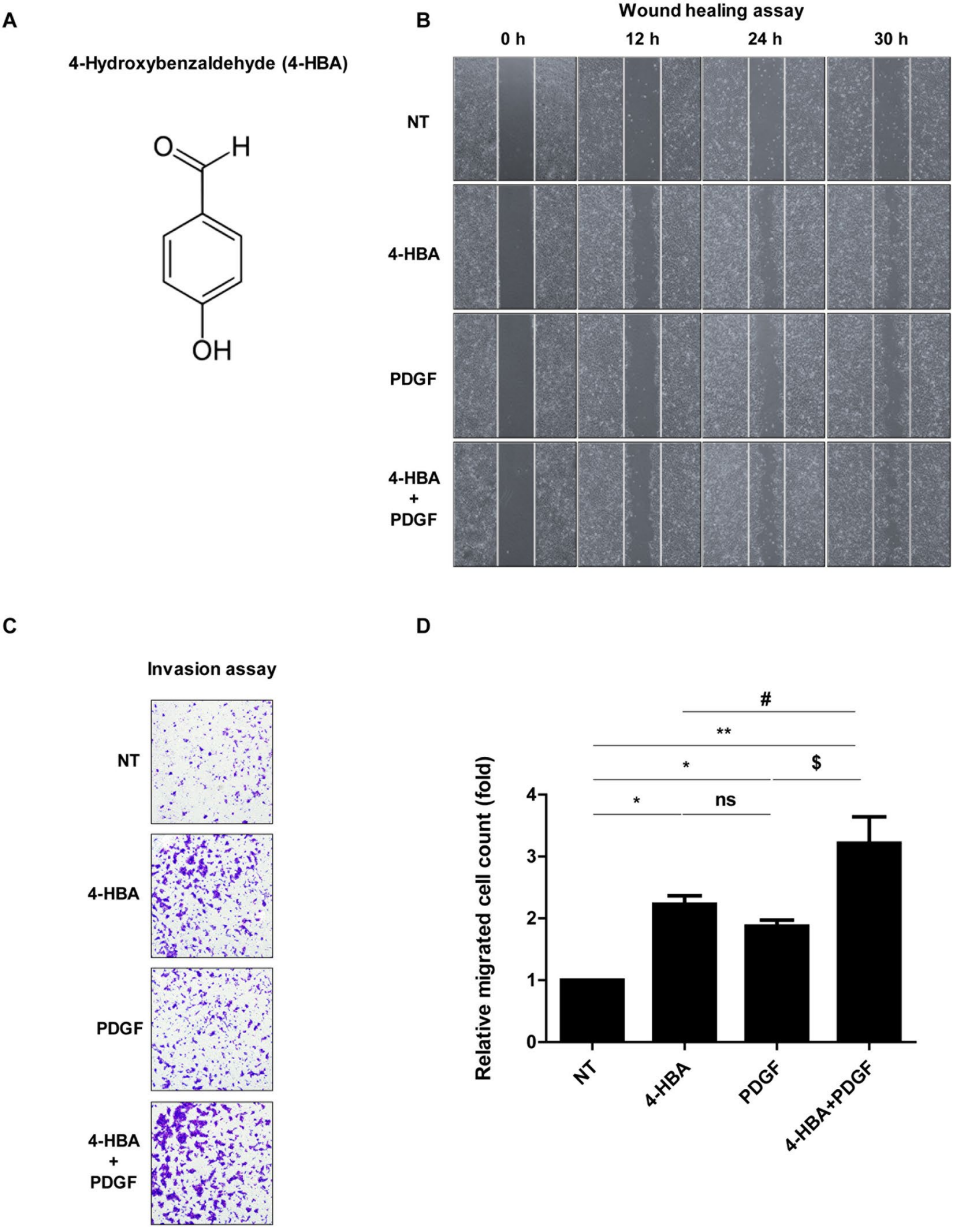
4-HBA induced keratinocyte migration. (**A**) Chemical structure of 4-hydroxybenzaldehyde (4-HBA). (**B**) *In vitro* scratch assay. HaCaT cells were treated with 4-HBA (0.1 mM), PDGF-BB (0.6 nM), or both 4-HBA and PDGF-BB or left untreated. (**C**) Invasion assay. Serum-starved HaCaT cells were treated with DMSO, 4-HBA (0.1 mM), PDGF-BB (0.6 nM), or both 4-HBA and PDGF-BB. (**D)** Relative migrated cell counts (n = 3; *P < 0.05 NT vs 4-HBA, *P < 0.05 NT vs PDGF-BB, **P < 0.01 NT vs 4-HBA + PDGF-BB, ns, non- significant 4-HBA vs PDGF-BB, ^#^ *P* < 0.05 4-HBA vs 4-HBA + PDGF-BB, $*P* < 0.05 PDGF-BB vs 4-HBA + PDGF-BB).

To study the effects of 4-HBA (Fig. 1A) on wound healing, we performed scratch wound-healing and invasion assays. HaCaT cells, an immortalized keratinocyte cell line, were incubated with 0.1 mM 4-HBA, PDGF-BB (0.6 nM as a positive control)^20–22^, or both, and migration was observed 12, 24, and 30 h after the scratch. As shown in Fig. 1B, 4-HBA treatment promoted wound closure as much as PDGF-BB. Transwell invasion assays also showed that 0.1 mM 4-HBA treatment enhanced the invasion of HaCaT cells by 2.5-fold compared with that in untreated cells. In HaCaT cells, 0.6 nM PDGF-BB treatment enhanced invasion by 1.6-fold compared with that in untreated cells. Therefore, 4-HBA treatment promoted HaCaT cell migration more effectively than PDGF-BB treatment (Fig. 1C and D). Moreover, as shown in Fig. 1B and C, the combination of 4-HBA and PDGF-BB showed synergistic effects by increasing cell migration and invasion by 3-fold compared with that in untreated cells. These results indicated that 4-HBA treatment enhanced the migration of keratinocytes.

Cell proliferation assays were then performed to exclude the possibility that the effects of 4-HBA on wound closure were due to increased cell proliferation. Treatment with 0.1 mM 4-HBA for 48 or 72 h in HaCaT cells slightly increased cell proliferation; however, the effects were not concentration-dependent or significant (Supp. Fig. 1A and B). Collectively, these data showed that 4-HBA strongly induced keratinocyte migration and invasion.

### 4-HBA induced cell migration via activation of focal adhesion kinase (FAK)/Src

Next, we investigated the mechanisms through which 4-HBA promoted HaCaT cell migration and invasion by 4-HBA treatment. In particular, we focused on focal adhesion signalling, as activation of FAK/Src is known to be involved in cell proliferation, invasion, and angiogenesis.

**Figure 2:**
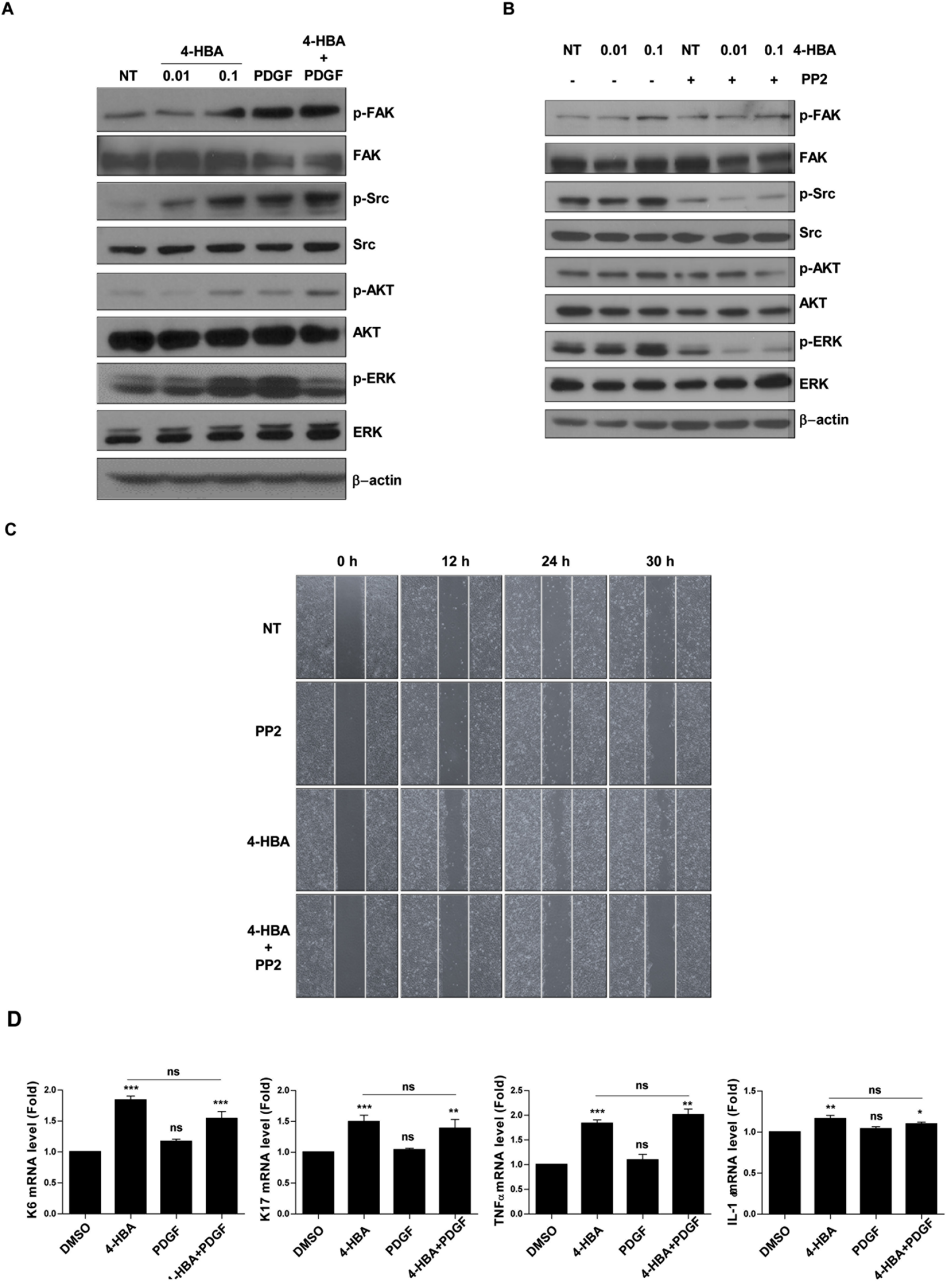
4-HBA induced cell migration via activation of various keratinocyte migration factors. (**A**) Western blot analysis assessing the effects 4-HBA, PDGF-BB, or combination treatment on HaCaT cells. Cells were treated with 0.01 mM 4-HBA, 0.1 mM 4-HBA, 0.6 nM PDGF-BB, or both. Lysates were evaluated by western blotting with the indicated antibodies. (**B**) HaCaT cells were pre-treated with DMSO or 5 μM PP2 (an Src inhibitor) for 2 h before treating with 0.01 mM or 0.1 mM of 4-HBA. Lysates were evaluated by western blotting with the indicated antibodies. (**C**) *In vitro* scratch assay. HaCaT cells were treated with DMSO, PP2 (5 μM), 4-HBA (0.1 mM), or both 4-HBA and PP2. (**D**) Effects of 4-HBA on the mRNA expression of genes encoding K6, K17, TNF-α, and IL-1α. Cells were treated either with 0.1 mM 4-HBA, 0.6 nM PDGF-BB or both 4-HBA and PDGF-BB, or left untreated for 8 h. The mRNA expression levels of each gene were normalized to that of β-actin mRNA. Each value is the mean ± SD of the three independent experiments. *P < 0.05, **P < 0.01, ***P < 0.001 versus DMSO group, ns, non- significant DMSO vs PDGF-BB, ns, non-significant 4-HBA vs 4-HBA + PDGF-BB.

We performed western blotting to determine whether 4-HBA affected FAK/Src signalling. As shown in Fig. 2A, 4-HBA induced the phosphorylation of FAK and Src in a concentration-dependent manner, suggesting that the effects of 4-HBA on cell migration and invasion may involve FAK/Src signalling. We further examined other major regulators of cell migration and survival and showed that 0.1 mM 4-HBA treatment increased the phosphorylation of extracellular signal-regulated kinase (ERK) and AKT (Fig. 2A). Next, we used PP2, a selective inhibitor of Src, to examine whether Src inhibition by PP2 would affect AKT and ERK phosphorylation in 4-HBA-treated HaCaT cells. As shown in Fig. 2B, 4-HBA induced the protein expression of p-FAK, p-Src, p-AKT, and p-ERK. Consistent PP2 treated-HaCaT cells exhibited no response to 4-HBA treatment (Fig. 2B). In line with western blot results, the rate of wound gap closure in PP2-treated HaCaT cells was not different from that of the control cells, even after 4-HBA treatment (Fig. 2C). These results indicate that FAK/Src-dependent activation of the AKT and ERK pathways was involved in 4-HBA-induced migration of HaCaT cells.

### 4-HBA enhanced the expression of various keratinocyte migration factors

K6 and K17 keratins, which are known to enhance the viscoelastic properties of migrating cells, are upregulated in migrating keratinocytes^23^, and their expression is regulated by growth factors within the wound environment^24^. Cytokines, such as interleukin (IL)-1 and tumour necrosis factor (TNF)-α, also modulate the migratory phenotypes of keratinocytes^25,26^. Thus, we next evaluated the expression of K6, K17, TNF-α, and IL-1in 4-HBA-treated HaCaT cells. We performed real-time polymerase chain reaction (PCR) experiments to measure the expression of keratinocyte migrating factors. As shown in Fig. 2D, the expression of K6 (1.76-fold), K17 (1.54-fold), TNF-α (1.55-fold), and IL-1α (1.3-fold) was increased in HaCaT cells treated with 0.1 mM 4-HBA compared with that in the untreated control group. PDGF-BB showed a slight increase, which was not statistically significant. However, since PDGF-BB treatment did not increase expression of K6, K17, TNFα, and IL-1α, the combination group was also not significantly effective compared with the 4-HBA (Fig. 2D). These data suggested that 4-HBA stimulated cell migration through upregulation of keratinocyte migration factors, such as TNF-α and IL-1.

### 4-HBA accelerated acute wound closure via keratinocyte migration in mice

**Figure 3:**
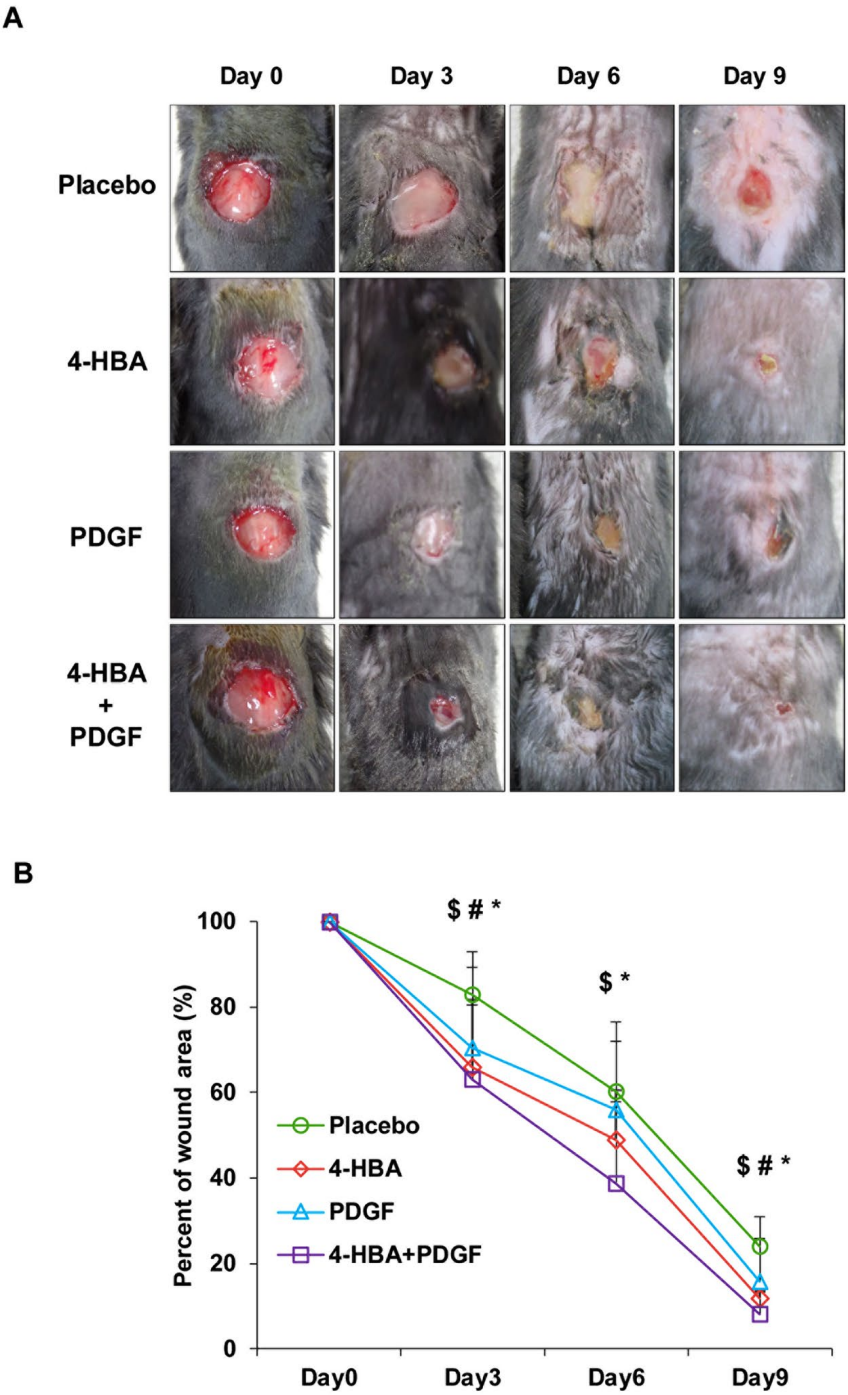
4-HBA accelerated acute wound closure in mice. (**A**) Full-thickness skin wounds (10 mm circle) in C57BL6 mice were treated with either placebo gel (5% CMC gel) or gel containing optimized concentrations of 4-HBA (0.66 mM), PDGF-BB (20 nM), or both 4-HBA and PDGF-BB. Images from one representative experiment are shown (n = 3 mice per group). (**B**) Measurements of placebo vs 4-HBA, PDGF-BB, or both 4-HBA and PDGF-BB in acute wound healing. Percentages of wound closure on days 0, 3, 6, and 9 after treatment with 4-HBA (0.66 mM), PDGF-BB (20 nM), and both 4-HBA and PDGF-BB versus placebo (mean ± SD). ^$^ *P* < 0.05, 4-HBA compared with placebo. ^#^ *P* < 0.05, PDGF-BB compared with placebo. *P < 0.05, 4-HBA + PDGF-BB compared with placebo.

Next, we created 10 mm circle wounds on the dorsal skin of C57BL6 mice to confirm the effect of 4-HBA in an *in vivo* system. 4-HBA, PDGF-BB, or a combination of 4-HBA and PDGF-BB were applied to the wounds, and the closure rate was measured every 3 days until day 9 (Fig. 3B). Representative images of each group are presented in Fig. 3A. Similar to the *in vitro* results, 4-HBA (34%) accelerated the wound closure more effectively than PDGF-BB (29.4%), even at a lower dose, on day 3. Moreover, the combination of 4-HBA and PDGF-BB resulted in 10% faster closure than 4-HBA treatment and 20% faster closure than PDGF-BB treatment on day 6 (Fig. 3B).

**Figure 4:**
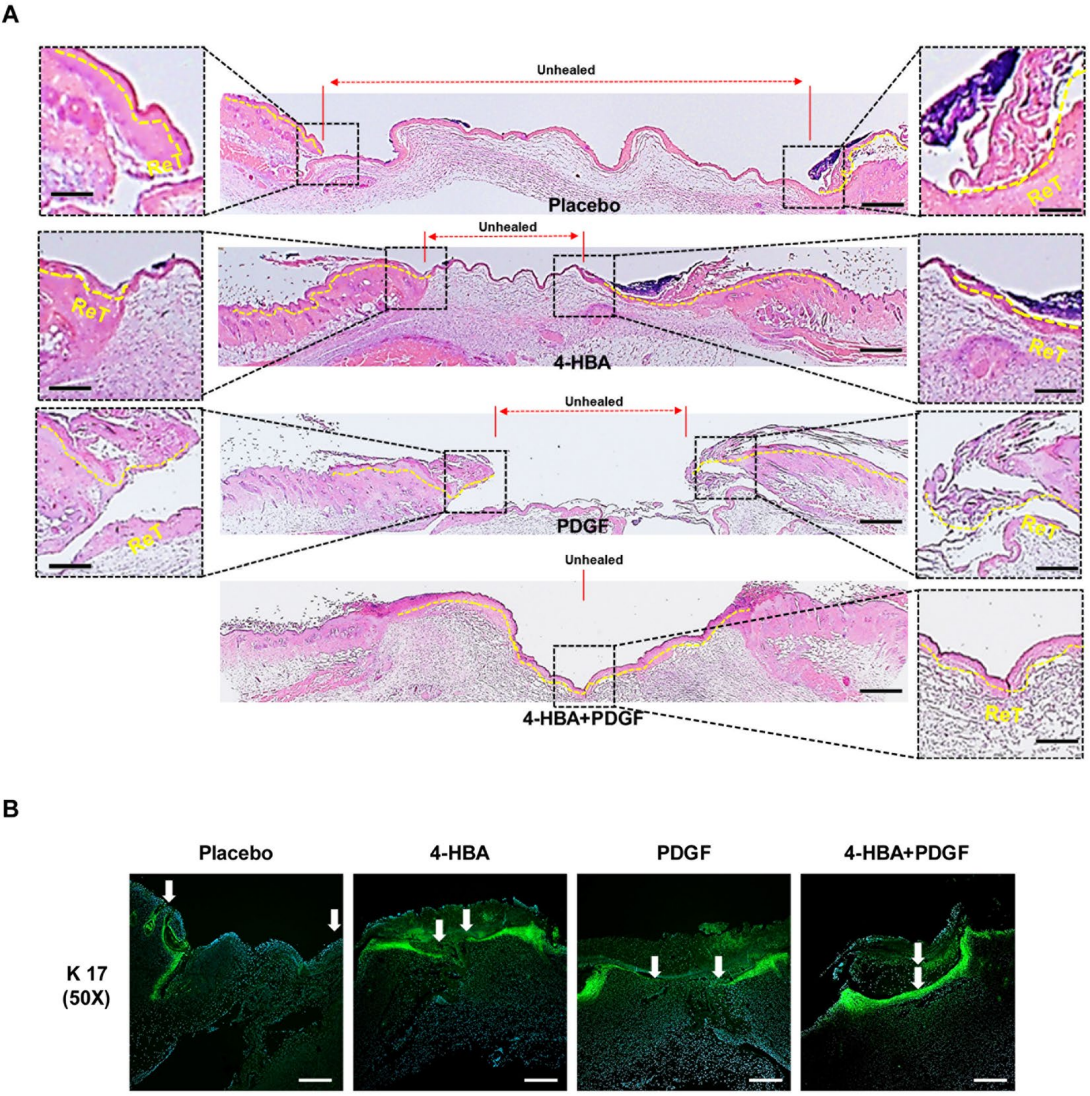
Effects of 4-HBA on wound healing *in vivo*. (**A**) 4-HBA promoted re-epithelialization of the wound. On day 6, wedge biopsies of full-thickness wounded skin (i.e., including a portion of unwounded skin) were stained with H&E and photographed under a light microscope. Independently photographed images with identical magnifications are shown. Red dotted lines indicate unhealed wound space. Yellow dotted lines mark the newly re-epithelialized epidermis. Red lines with arrows indicate unhealed skin areas. The fronts of newly re-epithelialized tongue (ReT) were enlarged, as shown in higher-magnification images. Scale bars: 0.2 mm (middle); 0.1 mm (left and right). (**B**) On day 9, wedge biopsies of full-thickness wounded skin were stained with anti-K17 and photographed under a fluorescence microscope. Independently photographed images with identical magnifications are shown. A green signal indicates newly migrating keratinocytes, and white arrows indicate the margins of migrating keratinocytes. DAPI (blue) was used for nuclear staining. Scale bar: 100 μm.

To closely examine the wounds, biopsies of each group from day 6 were subjected to haematoxylin and eosin (H&E) staining. As expected, 4-HBA treatment resulted in a smaller unhealed area than that of control group (Fig. 4A). Moreover, the wound area of the treated group showed a substantial increase in re-epithelialization. Interestingly, in the group treated with the combination of 4-HBA and PDGF-BB, the wound was completely closed.

Next, immunofluorescence staining (IF) was performed using anti-K17 antibodies to determine whether re-epithelialization by each drug treatment was due to keratinocyte migration^24^. As shown in Fig. 4B, keratinocyte migration increased in 4-HBA-treated wounds, and combined treatment with 4-HBA and PDGF-BB completely closed the wound with re-epithelialization. These results suggested that 4-HBA promoted acute wound healing via acceleration of keratinocyte migration and that the combination of 4-HBA and PDGF-BB had additive effects in promoting *in vivo* re-epithelialization.

### Combined treatment with 4-HBA with PDGF-BB as an angiogenesis stimulator *in vivo*

**Figure 5:**
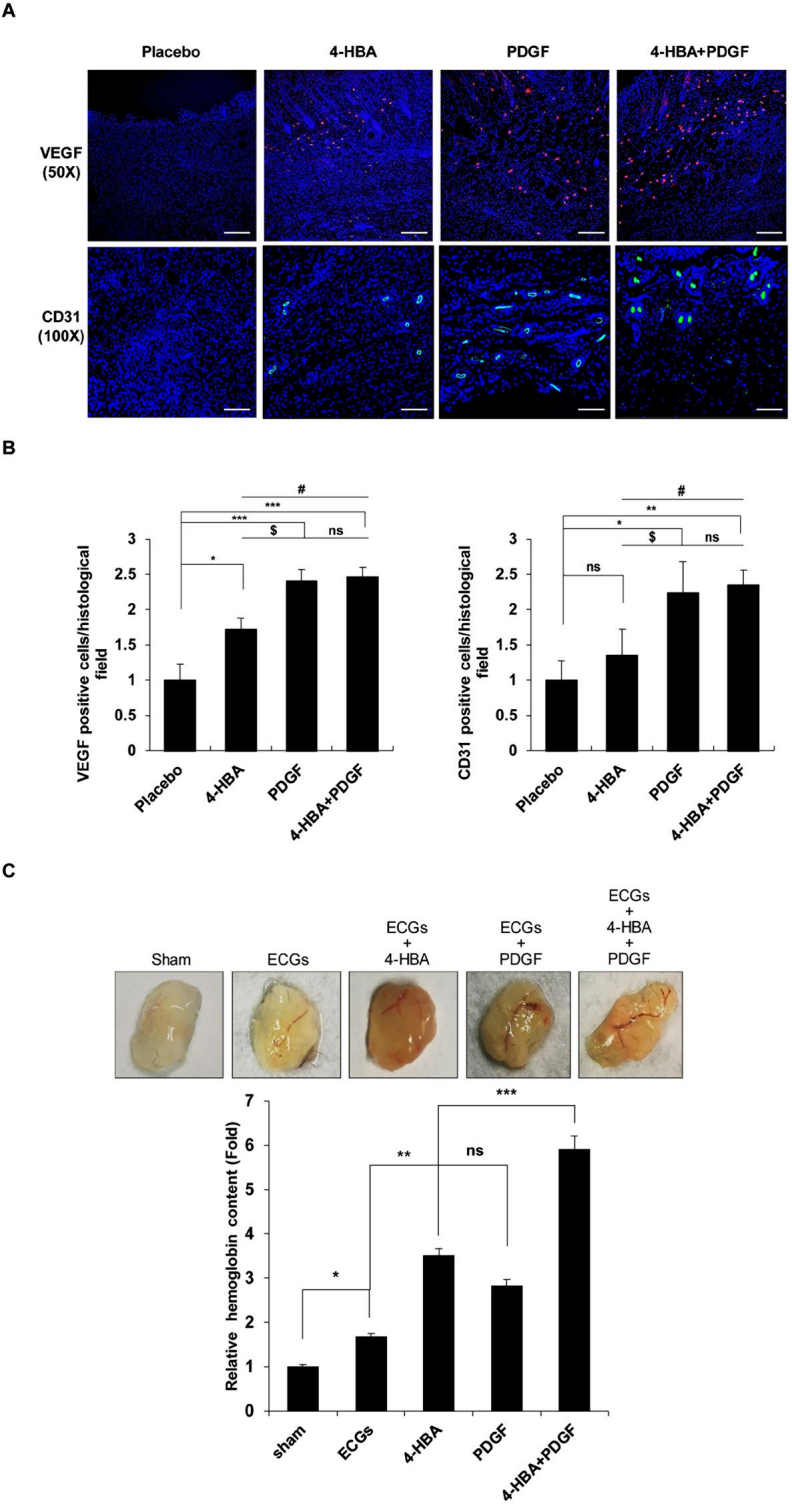
Combined treatment with 4-HBA and PDGF-BB enhanced angiogenesis. (**A**) On day 9, wedge biopsies of full-thickness wounded skin were stained with anti-VEGF or anti-CD31 antibodies and photographed under a fluorescence microscope. Red indicates VEGF expression, and green indicates CD31 (endothelial cells). DAPI (blue) was used for nuclear staining. Scale bar (VEGF): 100 μm. Scale bar (CD31): 50 μm (**B**) Quantitative analysis of VEGF (left) and CD31 (right) positive cells per histological field. Data were expressed as mean ± SD. (*P < 0.05 placebo vs 4-HBA, ***P < 0.001 placebo vs PDGF-BB, ***P < 0.001 placebo vs 4-HBA + PDGF-BB, ^$^ *P* < 0.05 4-HBA vs PDGF-BB, ^#^ *P* < 0.05 4-HBA vs 4-HBA + PDGF-BB, ns, non-significant PDGF-BB vs 4-HBA + PDGF-BB). (Right) CD31 immuno-fluorescence staining: (ns, non-significant placebo vs 4-HBA, *P < 0.05 placebo vs PDGF-BB, **P < 0.01 placebo vs 4-HBA + PDGF-BB, ^$^ *P*  < 0.05 4-HBA vs PDGF-BB, ^#^ *P* < 0.05 4-HBA vs 4-HBA + PDGF-BB, ns, non-significant PDGF-BB vs 4-HBA + PDGF-BB). (**C**) Representative Matrigel plugs were photographed (n = 3 in each group). Quantification of haemoglobin content was conducted using Matrigel plugs stained for infiltrating endothelial cells with anti-CD31 antibodies. *P < 0.05, sham vs ECGs, **P < 0.01, ECGs vs 4-HBA, ***P < 0.001 4-HBA vs 4-HBA + PDGF-BB, ns, non-significant 4-HBA vs PDGF-BB.

Angiogenesis, the process of new blood vessel formation, is a critical process in wound healing^27,28^. Since VEGF plays an important role in angiogenesis, we examined whether 4-HBA could increase the expression of VEGF in human dermal fibroblast (HDF) cells (Supp. Fig. 2A). In addition, we investigated the angiogenic activity of 4-HBA in an e*x vivo* model using a Sprout ring assay. Treatment with 4-HBA significantly promoted the growth of aortic sprouts compared with the serum control group. Consistent with this observation, sprout length was also significantly increased (Supp Fig. 2B), suggesting that 4-HBA may play an important role in stimulating angiogenic effects. To confirm the angiogenic effect of 4-HBA *in vivo*, full-thickness wound sections isolated from mice were stained with anti-VEGF and anti-CD31 antibodies as markers of endothelial cells and blood vessels, respectively. As shown in Fig. 5A, treatment with 4-HBA (0.66 mM) was less effective than treatment with PDGF-BB (20 nM), but increased the number of VEGF- and CD31-positive cells compared with that in control cells. In addition, the numbers of VEGF- and CD31-positive cells were significantly increased after combination treatment with 4-HBA and PDGF-BB.

We next performed *in vivo* Matrigel plug assays, which are often used for the rapid screening of potential pro-angiogenic compounds^29^. To examine the role of 4-HBA in new vessel formation, Matrigel containing endothelial cell growth supplement (ECG) treated with 4-HBA or PDGF-BB was subcutaneously injected into C57BL6 mice. After 7 days, haemoglobin content was measured in each group to quantify vascularization of the plugs (Fig. 5C). ECG induced a 1.67-fold increase in blood vessel formation compared with that in the sham control, and 4-HBA (0.66 mM) treatment increased vessel formation by 3.5-fold compared with that in the sham control. In plugs treated with 4-HBA, the haemoglobin content (3.5-fold) was higher than that in the control or PDGF-BB-treated group (2.8-fold), and the combination of 4-HBA (0.66 mM) and PDGF-BB (20 nM) increased the haemoglobin content by more than 5-fold compared with that in the sham control. These results demonstrate that 4-HBA promoted an increase in the formation of microvessels and that the combination of 4-HBA and PDGF-BB further promoted angiogenesis.

## Discussion

In this study, we compared the effects of 4-HBA and PDGF-BB on wound healing in mice and tested the ability of 4-HBA to promote wound healing. Our findings demonstrated that 4-HBA promoted the migration and invasion of human keratinocytes by inducing Src phosphorylation. Additionally, the effects of 4-HBA were greater than those of PDGF-BB, which is currently used for the treatment of acute wounds and diabetic ulcers. Our data provided preclinical evidence for the potential application of 4-HBA as an acute wound healing compound and showed that combination therapy of 4-HBA and PDGF-BB had synergistic effects.

The major cellular component of the epidermis is keratinocytes; which play important roles in barrier maintenance and re-epithelialization after injury^30^. To repair the injured epidermis, keratinocytes at the wound margin must to loosen themselves from the basal lamina and be flexible to migrate over the newly deposited matrix^31^. Src, a proto-oncogene encoding a membrane-associated, non-receptor protein tyrosine kinase, has roles in cell differentiation and proliferation^32,33^. Recent studies have shown that Src activity is inhibited by wound-induced keratin during keratinocyte migration and tissue repair^20^. Here, we demonstrated that 4-HBA induced cell migration through FAK/Src phosphorylation in a concentration-dependent manner. Src and FAK are cross-activating proteins^34^, and our findings demonstrated that 4-HBA induced the phosphorylation of both Src and FAK (Y397) in HaCaT cells. Autophosphorylation of activated FAK (Y397) recruits Src and generates an activated FAK/Src complex to enhance downstream signalling events, such as ERK and AKT phosphorylation^34^. Similar to PDGF-BB treatment, 4-HBA treatment significantly increased phosphorylation of ERK and AKT in keratinocytes, leading to keratinocyte cell migration. Keratinocyte migration is a complex process involving the continuous activation of kinases and cytokines. Our findings showed that treatment with 4-HBA increased mRNA expression of genes encoding IL-1 and TNF-α in keratinocytes. Moreover, the expression of K6 and K17 keratins in keratinocytes was also increased upon 4-HBA treatment. Taken together, our findings suggested that 4-HBA-induced Src and FAK phosphorylation promoted downstream signalling and keratinocyte migration.

Based on our previous study showing that 4-HBA promoted angiogenesis, we also expected 4-HBA to be effective in HDFs, which are important cells for neovascularization during wound healing^2,3^. However, 4-HBA was less effective than PDGF-BB in our *in vitro* wound healing, invasion assays, and cell adhesion in HDFs (Supp Fig. 1C). Moreover, 4-HBA treatment (0.1 mM) did not stimulate the phosphorylation of FAK, Src, or ERK in HDF cells (Supp Fig. 2A). In contrast, PDGF-BB significantly enhanced cell migration and invasion in HDF cells, and PDGF-BB significantly increased the phosphorylation of AKT and ERK and the expression of VEGF in HDF cells. These results indicated that the wound healing effects of 4-HBA were due to stimulation of keratinocyte migration rather than angiogenesis *in vitro*. Although 4-HBA was less effective to HDF cells, our *in vivo* Matrigel plug assays indicated that 4-HBA treatment enhanced new vessel formation and haemoglobin content compared with PDGF-BB treatment. In addition, 4-HBA treatment significantly increased sprout length in sprout ring assay (Supp Fig. 2B).These findings suggest that, 4-HBA accelerates wound healing process by promoting migration and angiogenesis.

Combination treatment with 4-HBA and PDGF-BB improved wound healing both *in vitro* and *in vivo* compared with 4-HBA or PDGF-BB alone. We attribute the synergistic effect of combination therapy to our results demonstrating that 4-HBA had more prominent effects on keratinocyte migration, whereas PDGF-BB was more effective in stimulating HDF cells and angiogenesis *in vivo*. Based on these results, the combination of 4-HBA and PDGF-BB potentially accelerated both early keratinocyte migration and late neovascularization. Further studies are required to identify the molecular mechanisms underlying these synergistic effects.

Owing to the diverse of natural herbal medicines, including their relative abundance, lower cost, and generally lower side effect profile, these medicines have been widely used for centuries to prevent and treat a variety of diseases^35^. In the present study, we assessed the effects of 4-HBA, an active constituent from *G*. *elata*, on wound healing. 4-HBA has antioxidant effects and is less expensive than PDGF-BB. However, the efficacy of 4-HBA for wound healing has not yet been reported. In accordance with the *in vitro* results, 4-HBA showed enhanced wound healing activity in mouse skin, and combination treatment with PDGF-BB increased wound healing by 17% compared with single treatment. Moreover, combination treatment was the most effective in accelerating keratinocyte migration, inducing the highest VEGF and CD31 expression, and enhancing haemoglobin content. Thus, 4-HBA was more effective than PDGF-BB, which is currently used clinically for the treatment of wound closure and diabetic limb ulcers, and the combination of PDGF-BB and 4-HBA showed excellent synergistic effects on wound healing, suggesting that this combined treatment could be an effective therapeutic approach.

In summary, our findings indicated that 4-HBA is a promising novel therapeutic agent for the treatment of acute wounds. Further research and clinical studies are required to confirm the safety and efficacy of this treatment strategy.

## Materials and Methods

### Compounds and antibodies

Recombinant Rat PDGF-BB was purchased from R&D Systems. 4-HBA was purchased from Sigma Aldrich (St. Louis, MO, USA). Antibodies against phospho (p)-FAK (Y397), p-SRC (Y416), SRC, p-AKT (S473), AKT, p-ERK (T202/204), ERK, and K17 were purchased from Cell Signaling Technology (Beverly, MA, USA). Anti-β-actin, VEGF and anti-platelet and endothelial cell adhesion molecule 1 (PECAM-1) antibodies were purchased from Santa Cruz Biotechnology, Inc. (Santa Cruz, CA, USA). Carboxymethylcellulose (CMC) sodium salt and kinase inhibitor PP2 were obtained from Sigma-Aldrich.

### Cell culture

HaCaT cells were cultured in Dulbecco’s modified Eagle’s medium (DMEM) containing 10% foetal bovine serum (FBS; Hyclone) and 1% Pen/Strep (Hyclone) at 37 °C in an atmosphere of 5% CO_2_. HDF cells were cultured in RPMI 1640 supplemented with 10% FBS and 1% Pen/Step. Depending on the type of experiment, cells were plated at different cell densities and on different culture dishes for various applications.

### Reverse transcription-PCR (RT-PCR)

Total RNA was extracted from HaCaT cells or HDF lysates with Isol RNA lysis reagent (5 Prime, Hilden, Germany), and cDNA was prepared using ReverTra Ace (Toyobo, Osaka, Japan) according to the manufacturer’s instructions.

### Western blotting

Preparation of whole-cell protein lysates and western blot analysis were carried out as described previously. Briefly, cells were then chilled on ice, washed twice with ice-cold phosphate-buffered saline (PBS), and lysed in a buffer (Cell Signaling Technology) containing 1 mM phenylmethylsulfonyl fluoride and 1x protease inhibitors (Sigma Aldrich). Protein concentrations were determined using a Bradford assay kit (Bio-Rad Laboratories, Hercules, CA, USA). Equal amounts of protein in cell lysates were separated by sodium dodecyl sulphate polyacrylamide gel electrophoresis (SDS-PAGE), transferred to membranes, and immunoblotted with specific primary and secondary antibodies, and the protein bands on the blots were detected using SuperSignal West Pico Chemiluminescent Substrate (Thermo Fisher Scientific), according to the manufacturer’s instructions.

### qRT-PCR analysis

Total RNA was extracted from HaCaT or HDF cell lysates using Isol-RNA lysis reagent (5 PRIME, Hilden, Germany), and cDNA was prepared using ReverTra Ace (Toyobo, Osaka, Japan), according to the manufacturer’s instructions. The following forward and reverse primers were used for amplification.

Tumour necrosis factor α (TNFα), 5′-ACCATGAGCACTGAAAGCAT-3′ and 5′- AGATGAGGTACAGGCCCTCT-3′; Vascular endothelial growth factor (VEGF), 5′-CTACCTCCACCATGCCAAGT-3′ and 5′-GCAGTAGCTGCGCTGATAGA-3′; Interleukin 1α (IL-1 α), 5′-CGCCAATGACTCAGAGGAAGA-3′ and 5′-AGGGCGTCATTCAGGATGAA-3′; Keratin 6, 5′-GAGCGGCCATGAAGAAGCT-3′ and 5′-TCCGCCATGCACCAACTTA-3′; Keratin 17, 5′-CATGCAGGCCTTGGAGATAGA-3′ and 5′-CACGCAGTAGCGGTTCTCTGT-3′; β-Actin, 5′-TGGCACCCAGCACAATGAAG-3′ and 5′-GACTCGTCATACTCCTGCTTGC-3′.

### MTS cell proliferation assay

For MTS assays, cultured cells were seeded into 96-well plates (3,000 cells/well). Twenty-four hours after seeding, serial dilutions of appropriate 4-HBA were added to the culture. Cell proliferation was measured after 48 h and 72 h following the manufacturer’s protocol. To measure cell proliferation, 20 μL of MTS labelling reagent was added to each well and incubated at 37 °C for 1 h. Absorbance was measured at 490 nm. Relative cell proliferation in the presence of drugs was normalized to DMSO-treated control cells after background subtraction.

### *In vitro* scratch assay

HaCaT cells were cultured for 24 h to achieve 100% confluence followed by starvation in serum-free DMEM. A 200 μL sterile pipette tip was used to make a scratch in the cell monolayer. Cells were then incubated in fresh medium containing 10% FBS for different times (0, 12, 24, and 30 h) at 37 °C in a 5% CO_2_ incubator. The scratch gap width at each time point in each treatment group was measured at four different positions and compared with the gap width at 0 h, which was arbitrarily set as 1.

### Cell adhesion assay

Collagen I-coated cell adhesion plates (Cell Biolabs, catalogue no. CBA-052) were allowed to warm to room temperature for 10 min. HaCaT and HDF cells were re-suspended in medium containing 0.2% BSA and either PP2, 4-HBA, PDGF or both PP2 and 4-HBA. In total, 1 × 10^6^ cells from each condition were transferred to individual wells and incubated for 1 h. Non- adherent cells were washed away, the remaining cells were stained and extracted, and the optical density was measured at 560 nm.

### *In vivo* wound healing assay

C57BL6 mice (6 weeks of age) were anesthetized using isoflurane prior to surgery. Full-thickness excision wounds (10-mm circle) were created by marking the area of the wound in the mid-back with a 10-mm punch, lifting the skin with a pair of a forceps, and excising the full-thickness skin along the lines with a pair of surgical scissors. Immediately after the surgery on day 0, the wounds were topically treated with 100 μL of either 5% CMC gel (placebo) or the same gel containing 4-HBA (0.66 mM), PDGF-BB (20 nM), or both 4-HBA and PDGF-BB. Each wound was covered with a bandage and a self-adherent wrap (Coban) to prevent desiccation and infection while the wound was exposed. Bandages and Cobans were changed every 3 days. Standardized digital photographs were taken of the wounds, with the same distance between the camera and pre-anesthetized animal. The relative wound area was calculated as wound area on day X/wound area on day 1. After sampling, mice were sacrificed by CO_2_ asphyxia. All animal experiments were performed according to all applicable Korean laws and were reviewed and approved by the Institutional Animal Care and Use Committee of Yonsei University Severance Hospital, Seoul, Korea (IACUC Approval No: 2016-01173) and were carried out in accordance with the approved guidelines by the IACUC.

### Invasion assay

The effects of 4-HBA on invasion in HaCaT cells were determined using a BD BioCoat Invasion assay system (BD Bioscience, Bedford, MA, USA) according to the manufacturer’s instructions. Briefly, HaCaT cells cultured in serum-free medium containing DMSO, 4-HBA, PDGF-BB, or both 4-HBA and PDGF-BB were seeded into the upper chambers of the assay system. Bottom wells were filled with complete medium. After incubation for 24 h, the cells in the upper chamber were removed, and the cells that had invaded through the Matrigel membrane were stained with crystal violet in PBS at room temperature for 1 h. The stained cells were photographed under a microscope.

### Sprout ring assay

Male Sprague Dawley Rats (100 g) were housed in a controlled environment and after one week of stabilization, under anaesthesia with Zoletil (30 mg/kg) and Rompun (10 mg/kg) by intraperitoneal injection, rats were sacrificed and thoracic aortas were obtained. Thoracic aortas were chopped into several pieces and placed on a Cell Culture insert (PICM03050) purchased from Millicell (Billerica, MA, USA) in a 6-well plate. EBM-2 serum media was added into the dish and incubated for 3 days. Next, 4-HBA was added, and the media was replaced every 2 days with substances. At day 7, aortas were photographed using an Olympus microscope at an appropriate magnification.

### H&E staining

On day 6 after wounding, mice were selected for H&E staining. Full-thickness skin covering the wounds and surrounding areas was excised, fixed in 4% paraformaldehyde for 24 h, embedded in paraffin, and then subjected to H&E staining. The unhealed wound width was defined as the distance between two opposite advancing edges of epidermal keratinocyte migration. The entire wound area was artificially reconstituted by overlapping multiple images.

### Immunofluorescence staining

On day 9 after wounding, samples were fixed in 4% paraformaldehyde, embedded in paraffin, and subjected to IF. Briefly, paraffin sections were cut to 4 μm thickness, rehydrated, blocked, and incubated with anti-K17 (1:100), anti-VEGF (1:200), and anti-PECAM-1 (1:200) primary antibodies overnight. The next day, the slides were washed with Tris-buffered saline containing 0.05% Tween 20, donkey anti-rabbit FITC or donkey anti-mouse Cy3 secondary antibodies (1:200; Jackson ImmunoResearch Laboratories, Inc) were added, and the slides were incubated for 1 h at room temperature. IF sections were mounted using Vectashield antifade mounting medium with DAPI. Images were captured using a Carl Zeiss Axio Imager M2 microscope.

### *In vivo* Matrigel plug assay

The Matrigel plug assay was performed as previously described^36^. Briefly, C57BL6 mice (Orient Technology, Seoul, Korea; weighing ~20 g; 7 weeks of age) were injected subcutaneously with 0.6 mL Matrigel containing saline, ECG supplement (354006; BD Bioscience), ECG + 4-HBA (0.1 mM), ECG + PDGF-BB (20 nM), or ECG plus 4-HBA (0.1 mM) and PDGF-BB (20 nM). After 7 days, the mice were anesthetized using Zoletil (30 mg/kg) and Rompun (10 mg/kg) by intraperitoneal injection. Next, the skins of the mice were pulled back to expose the Matrigel plug, which remained intact. The animals were then euthanized by carbon dioxide inhalation. Haemoglobin obtained from the Matrigel was measured using a Drabkin reagent kit 525 (Sigma-Aldrich) in order to qualify blood vessel formation. The concentration of haemoglobin was calculated in comparison to a known amount of haemoglobin assayed in parallel.

### Statistical analysis

Statistical calculations were performed with the using GraphPad Prism software (version 4.0.0; GraphPad Software, La Jolla, CA, USA). Each experiment was repeated at least three times. Statistical significance was analysed by one-way ANOVA with Tukey’s multiple comparison test.

## Electronic supplementary material

Supplementary Figure 1 and 2
