# Supplementary material for: 4-Hydroxybenzaldehyde accelerates acute wound healing through activation of focal adhesion signalling in keratinocytes
Source: Sci Rep. 2017 Oct 27;7:14192. doi: 10.1038/s41598-017-14368-y (PMC5660242; doi:10.1038/s41598-017-14368-y)
Supplement: Supplementary file 1 — Supplementary Figure 1 and 2 [file 41598_2017_14368_MOESM1_ESM.doc]

**4-Hydroxybenzaldehyde accelerates acute wound healing through activation of focal adhesion signalling in keratinocytes**

Chan Woo Kang^1,2^, Ye Eon Han^1,2^, Jean Kim^1,2^, Joo Heon Oh^2,3^, Yoon Hee Cho^2*^, Eun Jig Lee^2*^

^1^Brain Korea 21 PLUS Project for Medical Science, Yonsei University, Seoul Korea

^2^Endocrinology, Institute of Endocrine Research, College of Medicine, Yonsei University, Seoul, Korea

^3^Department of Biochemistry, Yonsei University, Seoul, Korea

*Corresponding authors.

Yoon Hee Cho : [wooriminji@gmail.com](mailto:wooriminji@gmail.com)

Eun Jig Lee : EJLEE423@yuhs.ac


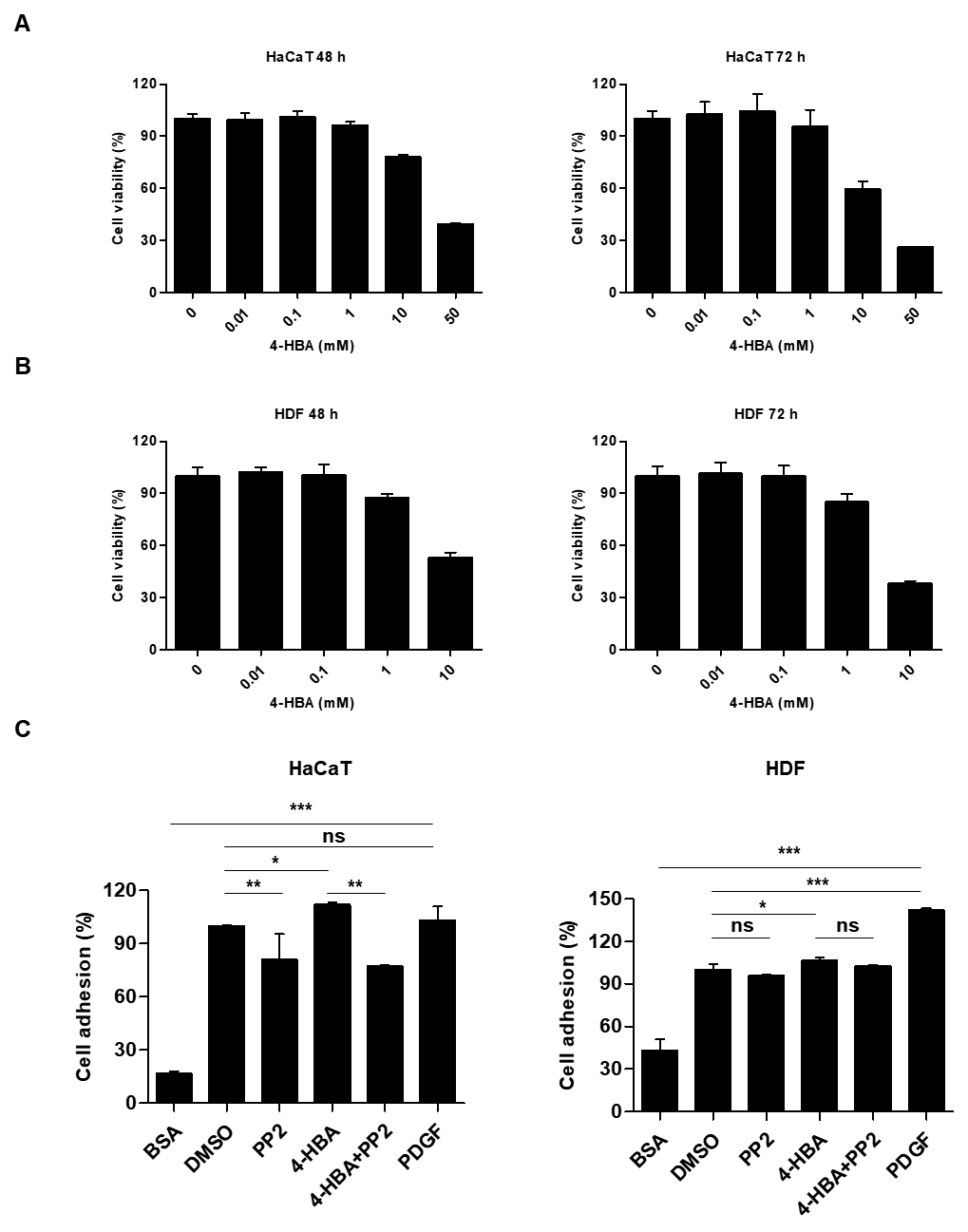


**Supplementary figure 1**

1. For MTS assay, HaCaT cells were treated with 4HBA (0, 0.01, 0.1, 1, 10, 50 mM) for 48 h (left) and 72 h (right).
2. For MTS assay, HDF cells were treated with 4HBA (0, 0.01, 0.1, 1, 10 mM) for 48 h (left) and 72 h (right).
3. (Left) Percentage of HaCaT adhesion to collagen I after a 1 h incubation with DMSO, PP2 (10 μM), 4-HBA (0.1mM), both PP2 and 4-HBA or PDGF (0.6 nM). BSA group is shown as negative control. (n = 4, *** *P* < 0.001 BSA vs DMSO, PP2, 4-HBA, 4-HBA+PP2, and PDGF, ** *P* < 0.01 DMSO vs PP2, ** *P* < 0.01 4-HBA vs 4-HBA+PP2, * *P* < 0.05 DMSO vs 4-HBA, ns, non-significant DMSO vs PDGF). (Right) Percentage of HDF adhesion to collagen I after a 1 h incubation with DMSO, PP2 (10 μM), 4-HBA (0.01mM), both PP2 and 4-HBA or PDGF (0.6 nM). BSA group is shown as negative control. (n = 4, *** *P* < 0.001 BSA vs DMSO, PP2, 4-HBA, 4-HBA+PP2, and PDGF, ns, non-significant DMSO vs PP2, ns, non-significant 4-HBA vs 4-HBA+PP2, * *P* < 0.05 DMSO vs 4-HBA, *** *P* < 0.001 DMSO vs PDGF).


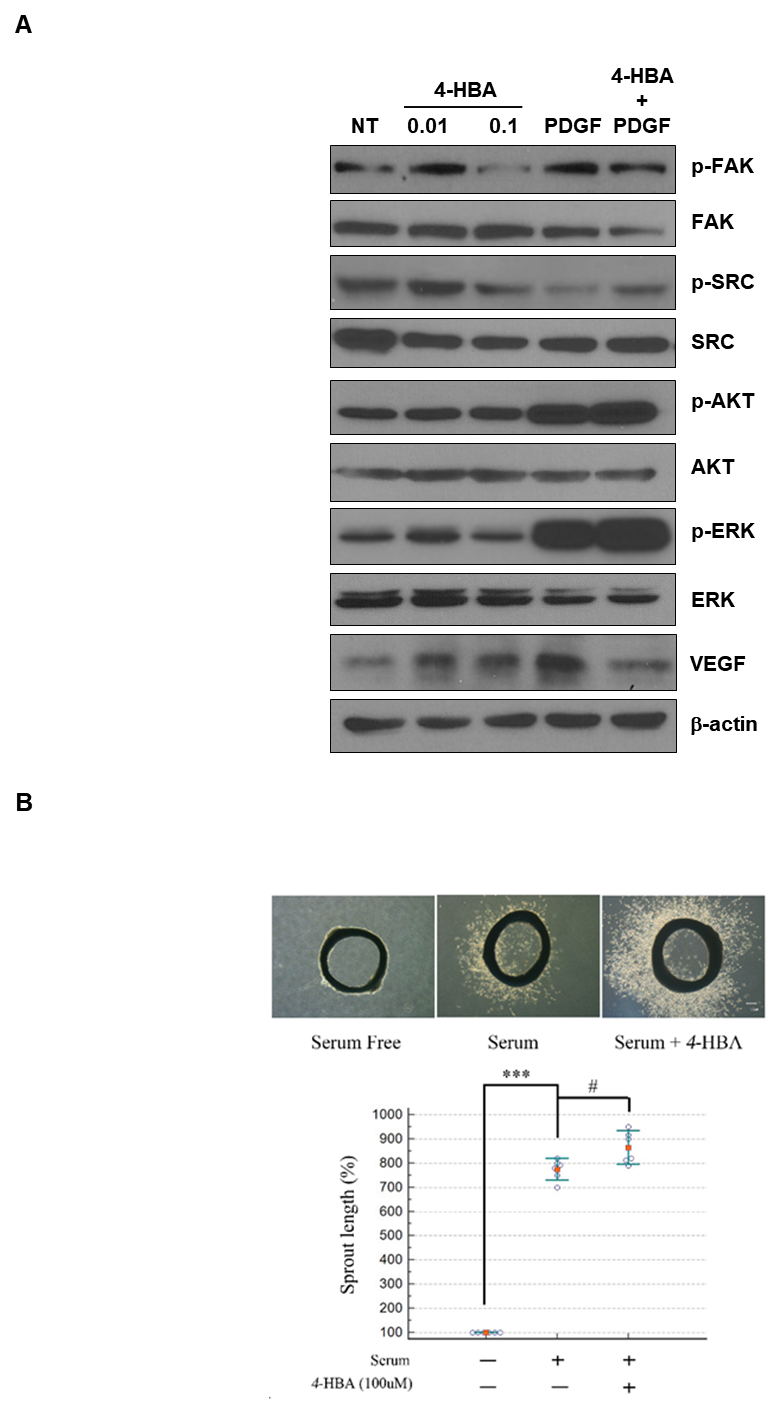


**Supplementary figure 2. Angiogenic effect of 4-HBA and PDGF-BB *in vitro, ex vivo*, and *in vivo***

1. Western blot analysis assessing the effect of 4HBA (0,01 and 0.1 mM), PDGF-BB (0.6 nM), or 4-HBA (0.1mM) +PDGF-BB (0.6 nM) treatment on HDF cells. Cells were treated with the indicated concentration of 4HBA, PDGF-BB, or combination. Lysates were evaluated by western blotting with the indicated antibodies.
2. (Up) Representative images of sprout ring assays are shown. (Down) Quantification of sprouting was measured using Scion Image software. Values represent the mean ± SEM of three experiments. *** indicates p < 0.001 compared to the serum-free group. # indicates p < 0.05 compared to the ECG group.
